# Obstetrician-Gynecologists’ knowledge of sickle cell disease screening and management

**DOI:** 10.1186/1471-2393-14-356

**Published:** 2014-10-14

**Authors:** Ijeoma C Azonobi, Britta L Anderson, Vanessa R Byams, Althea M Grant, Jay Schulkin

**Affiliations:** Department of Community Health and Preventive Medicine, Morehouse School of Medicine, Atlanta, GA USA; The American College of Obstetricians and Gynecologists, Washington, DC USA; Division of Blood Disorders, National Center on Birth Defects and Developmental Disabilities, Center for Disease Control and Prevention, Atlanta, GA USA

**Keywords:** Sickle cell disease, Physician practice patterns, Obstetrics

## Abstract

**Background:**

Although obstetrician/gynecologists (OB/GYNs) play an important role in sickle cell disease (SCD) screening and patient care, there is little information on knowledge of SCD or sickle cell trait (SCT) or related practices in this provider group. Our objective was to assess SCD screening and prenatal management practices among OB/GYNs.

**Methods:**

Twelve hundred Fellows and Junior Fellows of the American College of Obstetricians and Gynecologists (the College)^a^ were invited to complete a mailed survey, of which half (n = 600) belonged to the Collaborative Ambulatory Research Network.^b^ Participants answered questions regarding appropriate target patient groups for prenatal SCD screening, folic acid requirements, practice behaviors and adequacy of their medical school and residency training.

**Results:**

A total of 338 CARN members (56.3%) and 165 non-CARN members (27.5%) returned a survey. Of the 503 responders, 382 provided obstetric services and were included in the analyses. Forty percent of these respondents (n = 153) reported seeing at least 1 patient with SCD in the last year. Of these, 97.4% reported regularly screening people of African descent for SCD or SCT, whereas 52.9% reported regularly screening people of Mediterranean descent and 30.1% reported regularly screening people of Asian descent. Only 56.2% knew the correct recommended daily dose of folic acid for pregnant women with SCD. The proportion of respondents that rated training on SCD screening, assessment and treatment as barely adequate or inadequate ranged from 19.7% to 39.3%.

**Conclusions:**

The practice of many OB/GYNs who care for patients with SCD are not consistent with the College Practice Guidelines on the screening of certain target groups and on folic acid supplementation. There may be an opportunity to improve this knowledge gap through enhanced medical education.

## Background

Sickle cell disease (SCD) affects 80,000 to 100,000 people in the United States; approximately 3 million people are carriers of sickle cell trait (SCT) [1, 2]. Sickle cell is a complex disease for which successful management requires the coordination of care and health services from a multi-disciplinary team [3]. While great strides have been made in the pediatric arena, advances in SCD management have extended the lifespan of those affected and have increased the need for coordinated adult care [4, 5]. A study by Quinn et al. demonstrated that the risk of mortality in patients with SCD significantly increased shortly after transition into adult care [4]. The lack of specialists who are prepared to care for this population has been postulated as a cause of poor outcomes among these young adults [6]. Therefore, there has been a movement to equip primary care providers to care for this patient population.

Obstetrician/gynecologists (OB/GYNs) play an important role in SCD screening and adult patient care through their involvement with prenatal screening and pregnancy management. Consequently, OB/GYNs should be aware of the disease and basic principles of disease management. There is a paucity of information on provider knowledge and practice related to hemoglobinopathies internationally and in the United States, but what literature is available suggests that there are gaps in knowledge regarding newborn screening and the significance of trait status [7, 8]. However, most of the available literature focuses on provider knowledge regarding newborn screening, not prenatal screening, and does not specifically address obstetricians or provider knowledge of disease management [9]. The purpose of this study was to assess OB/GYN SCD-related screening and prenatal management practices as recommended by the American College of Obstetricians and Gynecologists (the College).

## Methods

### Study population

Eligible participants included Fellows and Junior Fellows of the College. Six hundred of these physicians were selected from the Collaborative Ambulatory Research Network (CARN), which includes practicing College members who volunteer to participate in research studies. Another 600 were selected from the general population of Fellows. Since the management practice questions pertained to prenatal care, we restricted our analysis to only those participants who reported that they provide obstetric services.

### Survey

The survey contained questions about the physicians’ demographics, training, practice characteristics and the practice population. Regarding screening practices, participants were asked about appropriate target groups for prenatal sickle cell screening. Participants were asked the recommended folic acid supplementation dose for this population and about their SCD referral and consultation practices. Additionally, participants were asked about sources they used for information on blood disorders and to rate their medical school and residency training experience regarding screening, assessment, and treatment of sickle cell disease. The survey was mailed to participants up to four times before they were considered a non-respondent. The study was reviewed and approved by the IRB at the American College of Obstetricians and Gynecologists. The study was of minimal risk and participation was voluntary. No identifying information was included in the data file.

### Statistical analysis

Statistical analysis was performed using SPSS version 16 (SPPS Inc., Chicago, IL, USA). Descriptive statistics such as percentages, means, and standard deviations were used to assess participant responses where appropriate.

## Results

Of the 1,200 eligible participants, 15 could not be reached and were excluded from the analysis. A total of 503 participants responded to the survey (overall response rate = 42.4%); 338 of these respondents were CARN members (56.3% response rate) and 165 were non-CARN members (27.5% response rate). Of the 503 responders, 382 provided obstetric services and were included in the analyses. No significant differences in age, sex, number of patients seen each week, number of patients seen each year, number of deliveries, number of surgeries, percent of patient races, primary care specialty, or residency of patients were found between CARN and non-CARN respondents; therefore the groups were combined for this analysis.

Table 1 shows participant demographics and practice characteristics. Fifty percent of participants were male. On average, participants had been in practice 16.6 years (standard deviation [SD] = 9.8) since residency. Fifty percent practice in an OB/GYN partnership or group setting. General OB/GYN was the reported primary medical specialty for 91.1% of participants. Respondents most frequently reported that their patients reside in urban, inner-city areas (41.9%). The average percent of African-American patients in each respondent’s practice was 14.5% (SD = 15.2%). The majority of participants (57.1%) reported that they had not provided care to any patients with SCD in the past year; however, of the 40.1% who had, the majority had provided care to 1–5 patients. Most participants reported that the College publications (82.7%) and obstetric journals (67.8%) were important sources of practice-relevant information regarding blood disorders.

**Table 1:** **Participant and practice characteristics (n = 382)**

***Characteristic***	***n***	***Mean***	***Percent or SD*** ^*^
**Age (mean)**	—	49	SD = 10.0
**Gender**			
Male	191		50.0
Female	191		50.0
**Primary practice location**			
Non-U.S.	12		3.2
Northeast	67		17.5
Midwest	92		24.1
Southwest	122		31.9
West	89		23.3
**Years in practice since residency (mean)**	—	16.6	SD = 9.8
**Current practice**			
Solo practice	49		12.8
Health Maintenance Organization	8		2.1
OB/GYN partnership/group	191		50.0
University full-time faculty and practice	53		13.9
Multi-specialty group	53		13.9
Other	28		7.3
**Primary medical specialty**			
General OB/GYN	348		91.1
Maternal-Fetal medicine	24		6.3
Obstetrics only	3		0.8
Reproductive endocrinology	1		0.3
Gynecology only	2		0.5
Other	4		1.0
**Residence of patients**			
Urban, inner city	160		41.9
Suburban	118		30.9
Mid-sized town	60		15.7
Rural	32		8.4
Military	8		2.1
Other	4		1.0
**Percent of patient race/ethnicity in practice (mean)**			
Non-Hispanic White	—	58.7	SD = 27.3
Hispanic	—	17.5	SD = 21.3
African-American	—	14.5	SD = 15.2
Native American	—	1.7	SD = 8.0
Asian/Pacific Islander	—	4.8	SD = 7.5
Other	—	1.7	SD = 7.3
**Patients seen each week (mean)**	—	89.4	SD = 41.0
**Number of SCD patients seen in the last year**			
None	218		57.1
1—5	126		33.0
6—10	17		4.5
11—15	4		1.0
16—20	5		1.3
21 or more	1		0.3

^*^Some columns do not total 100% because of missing responses.

SCD, sickle cell disease; SD, standard deviation.

Responses to questions regarding SCD screening and knowledge were restricted to the 153 respondents that reported seeing at least 1 patient with SCD in the last year. Ninety-seven percent of these respondents reported that they regularly screen people of African descent for SCD or SCT, whereas 52.9% regularly screen people of Mediterranean descent and 30.1% regularly screen people of Asian descent (Table 2). If the mother screens positive for SCD or SCT, 96.1% of respondents reported that efforts are made to screen the partner. If both parents screen positive for SCD or SCT, 89.5% of respondents routinely refer them to a genetic counselor.

**Table 2:** **Sickle cell disease (SCD) screening and management practices (n = 153)** ^*^

	***n (%)*** ^***†***^
**Which of the following ethnic groups are regularly screened as a part of prenatal care?** ^**‡**^	
All	29 (19.0)
**African descent** ^**§**^	**149 (97.4)**
**Mediterranean descent**	**81 (52.9)**
Middle Eastern descent	58 (37.9)
**Asian descent**	**46 (30.1)**
Northern European descent	32 (20.9)
Other	38 (24.8)
**What is the recommended daily folic acid intake for pregnant patients with SCD?**	
1 mg	42 (27.5)
2 mg	20 (13.1)
**4 mg**	**86 (56.2)**
6 mg	3 (2.0)

^*^Responses were restricted to only participants who provide obstetric services.

^†^Some columns do not total 100% because respondents were asked to check all that apply or because of missing responses.

^‡^Those who indicated “All” were included in totals for the individual ethnic groups.

^§^Responses in bold indicate correct responses for SCD, sickle cell disease.

Respondents were most likely to consult with a maternal-fetal medicine specialist (86.3%) or hematologist (83%) for patients with SCD. Only 56.2% of the respondents who had seen at least 1 patient with SCD in the last year knew the recommended daily intake of folic acid for pregnant patients with SCD (Table 2).

Regarding medical school and residency training, very few participants (0.3–2.1%) reported that their training on sickle cell screening, assessment or treatment was nonexistent (Table 3). However, 19.6% reported that training on SCD screening was barely adequate or inadequate, 28.5% reported that training on assessment of SCD was barely adequate or inadequate, and 39.3% reported that training on treatment of SCD was barely adequate or inadequate.

**Table 3:** **Description of sickle cell disease-related medical school and residency training (n = 382)**

	***n (%)*** ^*^				
	Comprehensive	Adequate	Barely adequate	Inadequate	Nonexistent
**SCD screening**	73 (19.1)	232 (60.7)	56 (14.7)	19 (5.0)	1 (0.3)
**SCD assessment**	55 (14.4)	212 (55.5)	83 (21.7)	26 (6.8)	5 (1.3)
**SCD treatment**	39 (10.2)	183 (47.9)	97 (25.4)	53 (13.9)	8 (2.1)

^*^Some responses do not total 100% because respondents did not provide an answer.

SCD, sickle cell disease.

## Discussion

OB/GYNs are crucial to the care of women with SCD for both prenatal screening and pregnancy management. Prenatal screening of new mothers and their partners can potentially identify people with the trait or the disease who would otherwise be missed by newborn screening, such as immigrants. Although improved management practices have reduced the adverse outcomes associated with pregnancy in women with SCD, there is still evidence from population-based studies that these women continue to be at increased risk of poor maternal and fetal outcomes such as preeclampsia, infection, preterm labor, fetal growth restriction, and fetal death [10–12]. As a consequence of these risks, healthcare providers that encounter these patients need to have a foundation of knowledge on SCD and its management in order to provide appropriate care for this population [13, 14].

Nonetheless, most studies regarding provider knowledge of SCD have focused on assessing knowledge of newborn screening, which many prenatal care providers believe falls under the purview of pediatricians [15, 16]. To our knowledge, this is the first study to assess the knowledge of sickle cell disease prenatal screening and related practice patterns among OB/GYNs.

The College has established guidelines for the management of hemoglobinopathies in pregnancy, which specify that women of African, Mediterranean, and Southeast Asian descent should be screened for hemoglobinopathies; that if found to be carriers, these women should be referred to genetic counseling; and that women with SCD should be prescribed 4 mg of folate supplementation daily [17]. In spite of this, we observed that while 97.4% of respondents regularly screened people of African descent, only 52.9% regularly screened people of Mediterranean descent, and 30.1% regularly screened people of Asian descent. Additionally, only 56.2% of respondents knew the correct amount of folate to prescribe to pregnant women with SCD. The College also recommends that couples at risk of having a child with a hemoglobinopathy be referred to a genetic counselor for a review of prenatal testing and reproductive options. We observed that 89.5% of respondents reported that they routinely referred couples to genetic counseling if both screened positive for SCT. This limited knowledge of SCD was consistent with the findings of a study of family health practitioners in Brazil, in which less than 75% correctly answered knowledge questions regarding SCD epidemiology, clinical manifestations and management [18].

The reason for the poor compliance with the College guidelines is unclear, considering that 82.7% of respondents reported that the College publications were an important source of information on blood disorders.

However, given that 19.6% to 39.3% of respondents believed training on SCD screening, assessment and treatment was barely adequate or inadequate, there may be an opportunity to enhance this knowledge via more robust training and medical education on SCD. There is evidence that interventions to increase provider knowledge regarding SCD can be effective [19]. Oyeku et al. [20] demonstrated that simple education strategies such as mailed materials or interactive seminars can increase provider knowledge of SCD newborn screening result management and increase provider confidence in providing newborn screening follow-up care. Our study suggests that an option for educating practicing OB/GYNs on SCD would be through publication of educational materials in OB/GYN specialty organization publications.

Our study was limited by several factors. CARN members, a group of physicians predisposed to participate in research studies, comprised the majority of respondents. The response rate of 42% was relatively low, but while this rate is comparable to other studies that included members of CARN [21, 22], it may have introduced responder bias and limited generalizability of the finding. Unfortunately, demographic information on non-respondents was not readily available for comparison, so the representativeness of the respondent sample could not be assessed. Also, the screening and management practice questions were restricted to the relatively small number of respondents who had provided care to 1 or more patients with SCD in the previous year. This could lead to bias in that providers who have seen patients with SCD may be primed to perform prenatal screening; therefore, these findings may have been an overestimation of the quality of management practices of the general population of OB/GYNs. Finally, since practice behaviors were assessed by self report, our assessment of these behaviors may have been subjective.

## Conclusions

In order to ensure appropriate delivery of healthcare for pregnant patients with sickle cell disease, it is of utmost importance that providers have a fundamental understanding of the disease and are aware of relevant professional management guidelines. Our study demonstrates a disconnect between the published College Guidelines on both appropriate target populations for prenatal SCD screening and recommendations for folic acid supplementation and the practice behaviors of OB/GYNs. If at-risk women and their partners are not screened, it is a missed opportunity to provide education and intervene when appropriate. Future efforts should focus on enhancing undergraduate and graduate medical education and disseminating information about SCD to practicing physicians through suitable channels.

## Endnotes

^a^The American College of Obstetricians and Gynecologists (the College) is a professional association of physicians specializing in obstetrics and gynecology. In order to become a Fellow or Junior Fellow, a board certified ob-gyn, whose professional activity is devoted to the practice of obstetrics and/or gynecology, needs to apply and be approved for Fellowship.

^b^The Collaborative Ambulatory Research Network is comprised of a group of practicing ACOG Fellows, who voluntarily participate in survey research on ambulatory care issues affecting Fellows of ACOG and their patients. Information from the studies is used to develop better informed educational strategies, and to disseminate information to physicians in those areas in which a knowledge deficit is apparent. Funding is provided by the cooperative agreement grant, UA6MC19010, through the U.S. Department of Health and Human Services, Health Resources and Services Administration, Maternal and Child Health Research Program.
